# Lidocaine-Based Derivatives for the Treatment of Staphylococcal Enterotoxin B-Induced Chronic Rhinosinusitis

**DOI:** 10.3390/ijms26178137

**Published:** 2025-08-22

**Authors:** Seung-Heon Shin, Mi-Kyung Ye, Mi-Hyun Chae, Dong-Won Lee, Ahmed S. Aboraia, Abu-Baker M. Abdel-Aal, Wesam S. Qayed, Hend A. A. Abd El-wahab, Ola F. Abou-Ghadir, Tarek Aboul-Fadl

**Affiliations:** 1 Department of Otolaryngology-Head and Neck Surgery, School of Medicine, Daegu Catholic University, Daegu 42472, Republic of Korea; miky@cu.ac.kr (M.-K.Y.); leonen@hanmail.net (M.-H.C.); neck@cu.ac.kr (D.-W.L.); 2 Department of Medicinal Chemistry, Faculty of Pharmacy, Assiut University, Assiut 71526, Egypt; ahmed.mohamed15@pharm.aun.edu.eg (A.S.A.); wesam.qayed@aun.edu.eg (W.S.Q.); hendaboel-maged@aun.edu.eg (H.A.A.A.E.-w.); fadl@aun.edu.eg (T.A.-F.); 3 Department of Pharmaceutical Organic Chemistry, Faculty of Pharmacy, Assiut University, Assiut 71526, Egypt; abubaker.elsayed@pharm.aun.edu.eg (A.-B.M.A.-A.); olaghadir@aun.edu.eg (O.F.A.-G.)

**Keywords:** chronic rhinosinusitis, mouse model, lidocaine, inflammation, cytokine

## Abstract

Lidocaine exhibited anti-inflammatory and immunomodulatory properties. This study aimed to investigate the anti-inflammatory effects of the lidocaine-derived analogs, EI137 and EI341, in a Staphylococcal enterotoxin B (SEB)-induced chronic rhinosinusitis (CRS). A CRS model was established using BALB/c mice via intranasal instillation of SEB. EI137 and EI341 were administered intranasally at 0.5 μg/g and 5 μg/g, respectively. Nasal symptoms and interleukin (IL)-4, IL-10, interferon (IFN)-γ, and tumor necrosis factor (TNF)-α levels in the nasal lavage fluid (NLF) were assessed. The reverse-transcription polymerase chain reaction was used to identify IFN-γ, IL-4, IL-10, and their transcription factors in the sinonasal mucosa. Histological changes were performed to assess inflammatory cell infiltration, epithelial thickness, and mucus-producing cells. SEB induced significant increases in IL-4, IL-10, and TNF-α levels in NLF and sinonasal mucosa, along with marked inflammatory cell infiltration. Intranasal EI137 and EI341 administration significantly reduced Th2 cytokine and its transcription factor, inflammatory cell infiltration, and mucus-producing cell numbers in the sinonasal mucosa. Further, EI137 suppressed Th1 cytokines, whereas EI341 enhanced Th1 responses. Both compounds promoted regulatory T cell responses, as evidenced by increased IL-10 and Foxp3 mRNA expression. EI137 and EI341 demonstrated potent local anti-inflammatory effects in a SEB-induced CRS model by modulating Th2 and Treg responses. EI137 suppressed Th1 inflammation, whereas EI341 enhanced it. These results indicate that EI137 and EI341 are promising topical agents for Th2-dominant inflammatory diseases, with distinct effects on Th1 immune responses.

## 1. Introduction

Chronic rhinosinusitis (CRS) is a chronic inflammatory disease of the sinonasal mucosa with various causative factors such as allergens, bacteria, fungi, and viruses. CRS is categorized into three endotypes based on T-helper (Th) cytokine expression and is classified as eosinophilic or non-eosinophilic CRS according to the dominant inflammatory cell types [1]. Type 2 or eosinophilic CRS is more prevalent in Western countries and has a poorer prognosis compared to type 1 and type 3, or non-eosinophilic CRS. Patients with eosinophilic CRS frequently suffer from thick viscous nasal discharge, olfactory dysfunction, and nasal polyposis. Staphylococcal enterotoxin B (SEB), fungi, and aeroallergens can induce peripheral blood and tissue eosinophilia by exacerbating type 2 airway inflammatory responses [1,2]. Recently, eosinophils have appeared as an important therapeutic target in recalcitrant CRS, and biologics have become a promising treatment option for managing eosinophilic airway mucosal inflammation [3].

Lidocaine has long been used as a local anesthetic agent, and several studies have investigated its possibility as a treatment agent for respiratory inflammatory disease due to its anti-inflammatory properties [4,5]. However, the discomfort associated with the local anesthetic effect and the risk of bronchoconstriction related to bronchial irritation limit its clinical application [6]. The tertiary amine group in lidocaine is essential for its local anesthetic activity; however, to overcome the limitations associated with this functional group, we synthesized a series of novel organic compounds based on the lidocaine scaffold, in which the tertiary amine group was removed [7]. These compounds have consistently exhibited anti-inflammatory and anti-allergic effects [8,9]. They possess immunomodulatory properties without inducing local anesthetic effects or bronchoconstriction [8,9]. However, lidocaine-based derivatives have yet to be translated into clinical practice despite their promising pharmacodynamic profile.

Eosinophils are multifunctional immune cells, and their increased numbers in the peripheral blood and sinonasal mucosa are a hallmark of eosinophilic CRS, characterized by excessive recruitment, activation, and prolonged survival of eosinophils [2]. Intranasal instillation of SEB has been established as a reliable method to induce eosinophilic CRS and nasal polyp formation in mice [10]. Among the synthetic molecules, lidocaine-derived organic compounds EI137 and EI341 have demonstrated strong inhibitory activity against interleukin (IL)-5-induced eosinophil functions, including activation, survival, and transcription factor expression [9]. In the current study, we aimed to assess the anti-inflammatory properties of EI137 and EI341 through intranasal administration in a SEB-induced eosinophilic CRS mouse model.

## 2. Results

### 2.1. Effect of Lidocaine-Derived Compounds on Sinonasal Symptoms

**Figure 1:**
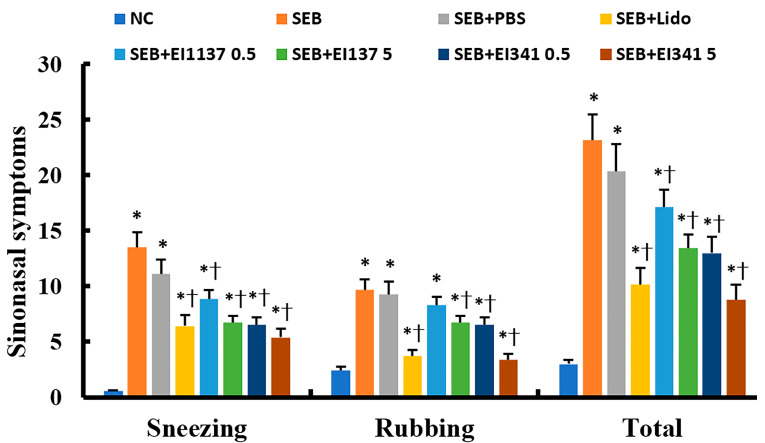
Effect of lidocaine analogs, EI137 and EI341, on sinonasal symptoms in the Staphylococcal enterotoxin B (SEB)-induced chronic rhinosinusitis mouse model. Intranasal administration of 5 μg/g of lidocaine (Lido) as well as 0.5 μg/g and 5 μg/g of both EI137 (137 0.5, 137 5) and EI341 (341 0.5, 341 5), respectively, significantly reduced sneezing and nasal rubbing. NC: negative control; PBS: phosphate-buffered saline; * *p* < 0.05 compared with NC; † *p* < 0.05 compared with SEB group (*n* = 8 mice per group).

After the final instillation of OVA, the mice were placed in an observation cage for 10 min to assess the number of sneezing and nasal rubbing motions. The frequency of sneezing and nasal rubbing was significantly increased in the SEB-induced CRS group than in the PBS-treated negative control mice. Intranasal administration of EI137 and EI341 at both 0.5 and 5 μg/g significantly and dose-dependently reduced the number of sneezing and nasal rubbing episodes. Similarly, intranasal instillation of lidocaine at 5 μg/g, which was administered as a positive control, also significantly suppressed these symptoms (Figure 1).

### 2.2. Effect of Lidocaine-Derived Compounds on Inflammatory Cell Profiles and Cytokine Levels in the Nasal Lavage Fluid (NLF)

**Figure 2:**
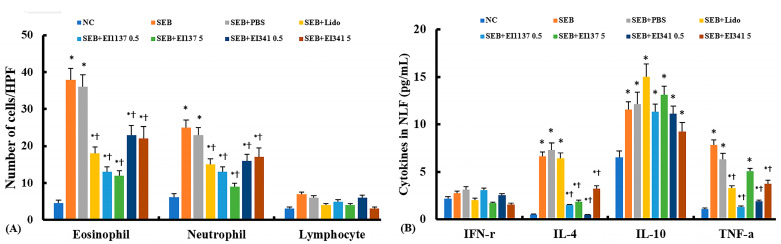
Effect of lidocaine analogs, EI137 and EI341, on inflammatory cell differentiation and cytokine levels in nasal lavage fluid in a Staphylococcal enterotoxin B (SEB)-induced chronic rhinosinusitis mouse model. (**A**) Eosinophil and neutrophil counts were significantly reduced by intranasal administration of 5 μg/g of lidocaine (Lido), as well as 0.5 μg/g and 5 μg/g of EI137 (137 0.5, 137 5) and EI341 (341 0.5, 341 5). (**B**) Both doses of EI137 and EI341 significantly suppressed IL-4 and TNF-α levels, whereas IL-10 levels remained unaffected. NC: negative control; PBS: phosphate-buffered saline; * *p* < 0.05 compared with NC; † *p* < 0.05 compared with SEB (*n* = 8 mice per group).

The number of eosinophils and neutrophils was significantly increased in the NLF of the CRS mouse model (eosinophil: 38.5 ± 9.6; neutrophil: 25.7 ± 7.3) compared with the negative control mice (4.6 ± 1.2 and 6.2 ± 0.9, respectively). Intranasal administration of EI137 and EI341 at 0.5 μg/g and 5 μg/g, respectively, significantly reduced eosinophil and neutrophil counts in the NLF. EI137 demonstrated a greater reduction in inflammatory cell counts than EI341, although this was not statistically significant (Figure 2A).

IL-4, IL-10, and TNF-α levels in NLF were significantly increased in the CRS mouse model (IL-4: 6.7 ± 1.4 pg/mL; IL-10: 11.6 ± 3.2 pg/mL, and TNF-α: 7.8 ± 3.1 pg/mL) compared with the negative control mice (0.5 ± 0.1 pg/mL, 6.6 ± 1.7 pg/mL, and 1.1 ± 0.4 pg/mL, respectively). Intranasal administration of EI137 and EI341 at both 0.5 μg/g and 5 μg/g significantly reduced IL-4 and TNF-α levels in NLF. The 0.5 μg/g dose of the lidocaine analogs tended to suppress cytokine levels more effectively than the 5 μg/g dose, although this was not statistically significant. However, the statistical analysis showed that the lidocaine analogs did not significantly affect the IL-10 level in NLF (Figure 2B).

### 2.3. Effect of Lidocaine-Derived Compounds on Cytokine and Their Transcription Factor mRNA Expression in the Sinonasal Mucosa

**Figure 3:**
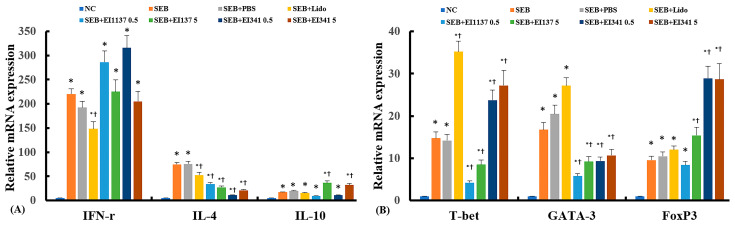
Effect of lidocaine analogs, EI137 and EI341, on the mRNA expression of cytokine IFN-γ, IL-4, IL-10 (**A**), and the transcription factors T-bet, GATA-3, and Foxp3 (**B**) in the sinonasal mucosa of a Staphylococcal enterotoxin B (SEB)-induced chronic rhinosinusitis mouse model. Intranasal administration of EI137 and EI341 at both 0.5 μg/g and 5 μg/g (137 05 and 137 5, 341 0.5, and 341 5) significantly suppressed IL-4 and GATA-3 mRNA expression in the sinonasal mucosa. NC: negative control; PBS: phosphate-buffered saline; Lido: lidocaine; * *p* < 0.05 compared with NC; † *p* < 0.05 compared with SEB (*n* = 5 mice per group).

Real-time reverse-transcription polymerase chain reaction (RT-PCR) was performed to assess the effect of intranasal administration of the lidocaine-derived compounds, EI137 and EI341, on mRNA expression of T helper (Th)-related cytokines and transcription factors in the sinonasal mucosa. The mRNA expression levels of IFN-γ, IL-4, IL-10, and their respective transcription factors were significantly increased in the SEB-induced CRS mouse model. Intranasal administration of both 0.5 μg/g and 5 μg/g of EI 137 and EI341 significantly suppressed the expression of the Th2 cytokine IL-4 and its transcription factor GATA-3. The mRNA expression of Th1 cytokine IFN-γ was not significantly affected by either compound (*p* > 0.05); however, EI137 decreased and EI341 increased the expression of its transcription factor T-bet. SEB-induced IL-10 mRNA expression was increased after administrating 5 μg/g of both EI137 and EI341. Similarly, 5 μg/g of EI137 and both 0.5 μg/g and 5 μg/g of EI341increased the expression of the IL-10-associated transcription factor FoxP3 (Figure 3).

### 2.4. Effect of Lidocaine-Derived Compounds on SEB-Induced Splenocyte Activation

**Figure 4:**
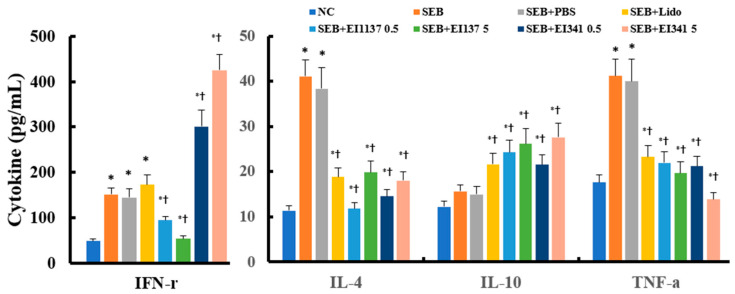
Effect of lidocaine analogs, EI137 and EI341, on IL-4, IL-10, IFN-γ, and TNF-α production by splenocytes in a Staphylococcal enterotoxin B (SEB)-induced chronic rhinosinusitis mouse model. Intranasal administration of EI137 and EI341 at both 0.5 μg/g and 5 μg/g (137 05, 137 5, 341 0.5, and 341 5) significantly suppressed SEB-induced IL-4 and TNF-α production compared with splenocytes from the untreated mouse model. NC: negative control; PBS: phosphate-buffered saline; Lido: lidocaine; * *p* < 0.05 compared with NC; † *p* < 0.05 compared with SEB (*n* = 8 mice per group).

To evaluate whether intranasal administration of lidocaine-derived compounds EI137 and EI341 elicited systemic immune responses, splenocytes were exposed to SEB stimulation. After 72 h incubation, concentrations of IL-4, IL-10, IFN-γ, and TNF-α in culture supernatants were determined using ELISA. Splenocytes isolated from the SEB-induced CRS mouse model demonstrated significantly increased IFN-γ, IL-4, and TNF-α production after SEB stimulation. However, SEB stimulation did not affect IL-10 production. Intranasal administration of EI137 and EI341 at both 0.5 μg/g and 5 μg/g significantly inhibited SEB-induced IL-4 and TNF-α production. Interestingly, EI137 significantly suppressed SEB-induced IFN-γ production, whereas EI341 significantly enhanced IFN-γ production. EI137 and EI341 increased IL-10 production in response to SEB stimulation. Lidocaine significantly suppressed SEB-induced IL-4 and TNF-α production and enhanced IL-10 production, but did not affect SEB-induced IFN-γ production (Figure 4).

### 2.5. Effect of Lidocaine-Derived Compounds on Histological Changes in the Sinonasal Mucosa

**Figure 5:**
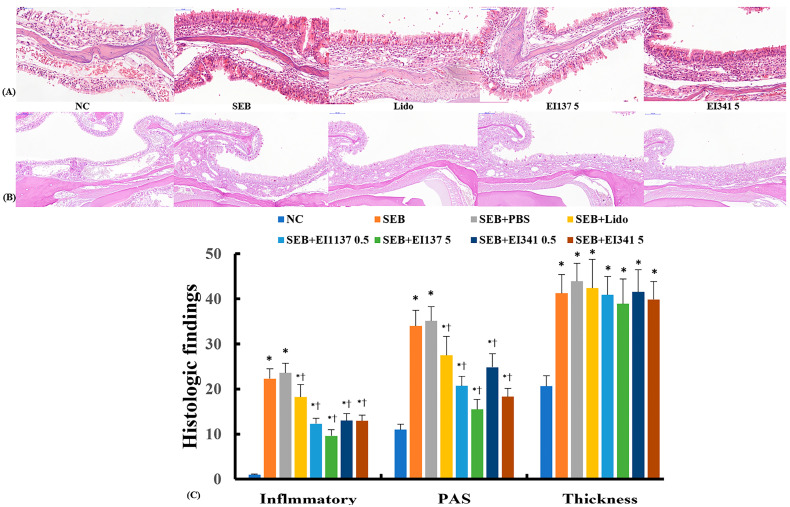
Effect of lidocaine analogs, EI137 and EI341, on the histologic characteristics of the sinonasal mucosa in a Staphylococcal enterotoxin B (SEB)-induced chronic rhinosinusitis (CRS) mouse model. The CRS model demonstrated increased inflammatory cell infiltration and periodic acid–Schiff (PAS)-positive mucus-producing cells, both of which were significantly reduced by intranasal administration of EI137 and EI341 at both 0.5 μg/g and 5 μg/g (137 0.5, 137 5, 341 0.5, and 341 5). However, Epithelial thickness was not affected by treatment with the lidocaine analogs (**C**). (**A**) Representative images of hematoxylin and eosin-stained tissues (×20); (**B**) Representative images of PAS-stained sinonasal mucosa (×20). NC: negative control; PBS: phosphate-buffered saline; Lido: lidocaine; * *p* < 0.05 compared with NC; † *p* < 0.05 compared with SEB (*n* = 5 mice per group).

The sinonasal mucosa of the SEB-induced CRS mice demonstrated significant inflammatory cell infiltration (22.3 ± 4.8), the number of PAS-positive mucus-producing cells (34.0 ± 8.1), and epithelial thickness (41.3 ± 5.7 μm) compared with the negative control mice (12.3 ± 0.3, 11.3 ± 3.7, and 20.7 ± 3.2 μm, respectively). Intranasal administration of EI137 and EI341 at both 0.5 μg/g and 5 μg/g significantly reduced inflammatory cell infiltration and the number of mucus-producing cells. However, the epithelial thickness was not affected by lidocaine analog treatment. Similarly, intranasal instillation of lidocaine significantly suppressed inflammatory cell infiltration and mucus-producing cells in the sinonasal mucosa, but did not alter SEB-induced epithelial thickening (Figure 5).

## 3. Discussion

Lidocaine, which is a local anesthetic agent, is known to possess anti-allergic, anti-inflammatory, and immunomodulatory effects [4,5]. In this study, we used lidocaine-derived organic compounds, EI137 and EI341, which exhibited an anti-eosinophilic effect with anti-allergic properties [9,11]. Intranasal instillation of EI137 and EI341 significantly reduced inflammatory cell infiltration in the sinonasal mucosa and NLF, as well as type 2 inflammation in the SEB-induced CRS mouse model. EI137 markedly suppressed systemic Th1 and Th2 inflammation, whereas EI341 improved systemic Th1 inflammation while suppressing Th2 responses. These anti-inflammatory effects were comparable to or even stronger than those observed with the intranasal administration of lidocaine.

SEB induced eosinophilic type 2 CRS with underlying allergic inflammation in BALB/c mice [9]. BALB/c mice are widely used in various immunological studies because they demonstrate both Th1 and Th2 immune-mediated airway inflammatory responses more distinctly than other mouse strains [12,13]. This SEB-induced eosinophilic CRS model has been used to evaluate the anti-inflammatory effects of various chemical and biological agents [14,15]. The combination of OVA and SEB has markedly exacerbated sinonasal mucosal inflammation and significantly increased the recruitment of eosinophils and neutrophils. These inflammatory cell infiltrations are associated with increased proinflammatory cytokine TNF-α levels, which promote leukocyte infiltration and local inflammation by inducing additional inflammatory cytokine production [16]. In this study, intranasal instillation of lidocaine analogs significantly suppressed TNF-α levels in NLF, along with decreased eosinophil and neutrophil counts. Eosinophilic components, such as Charcot–Leyden crystals, further exacerbate neutrophilic inflammation by promoting neutrophil infiltration in CRS [17]. In severe CRS, tissue neutrophilia and neutrophilic inflammation are frequently caused by the products of eosinophilic inflammation [17,18]. In this study, SEB-induced CRS mice demonstrated increased IL-4 mRNA expression and prominent inflammatory cell infiltration in the sinonasal mucosa, indicating that SEB effectively induces a strong type 2 sinonasal inflammation. Intranasal administration of EI137 and EI341 significantly suppressed Th2 cytokine expression in NLF, reduced IL-4 mRNA and its transcription factor expression in the sinonasal mucosa, and decreased both eosinophil and neutrophil counts in NLF as well as inflammatory cell infiltration in the sinonasal mucosa. These findings indicate that lidocaine analogs may effectively control SEB-induced sinonasal mucosal inflammation and represent a promising therapeutic candidate for severe recalcitrant CRS treatment.

To assess the systemic effects of the intranasal instillation of lidocaine analogs, we investigated SEB-induced cytokine production from splenocytes isolated from CRS mice. SEB stimulation significantly increased IFN-γ, IL-4, IL-10, and TNF-α production by splenocytes from SEB-induced CRS mice. Among these cytokines, Th2 cytokine IL-4 and TNF-α production were significantly decreased in mice treated intranasally with EI137 and EI341. Interestingly, Th1 cytokine IFN-γ production was decreased by EI137 but increased with EI341, indicating a distinct immunomodulatory effect of the lidocaine analogs. EI137 and EI341 did not affect IFN-r mRNA expression in the sinonasal mucosa. However, EI137 suppressed the expression of T-bet, which is a Th1 transcription factor, whereas EI341 upregulated T-bet expression, which further supports the differential effects of these compounds on the Th1 response. These results indicate that EI137 and EI341 may have distinct immunomodulatory properties in regulating sinonasal mucosal inflammation. EI137 suppressed Th1 inflammatory responses, whereas EI341 enhanced Th1 responses in SEB-induced CRS. EI137 and EI341 are positional isomers, meaning they have the same chemical components but differ in the arrangement of chloro and methyl groups, which may account for their distinct effects on Th1 immune responses through different electronic properties. To elucidate the opposing immunomodulatory effects of EI137 and EI341 on Th immune responses, we performed a molecular docking analysis of lidocaine analogs with STAT-1 (Method S1). The computational modeling demonstrated that, although EI137 and EI341 possess similar binding affinities, they exhibited opposite biological activities (Result S1 and Figures S1 and S2). These findings suggest that the STAT-1-inhibitory binding of EI137 accounts for its suppression of IFN-γ production and Th1 responses, whereas the non-competitive binding of EI341 is consistent with its Th1-enhancing activity. IL-10, which is a key immunoregulatory anti-inflammatory cytokine involved in maintaining immune homeostasis in the sinonasal mucosa, was increased in splenocytes from mice treated with EI137 and EI341 when stimulated with SEB. Consistently, these lidocaine analogs enhanced Treg cytokine IL-10 mRNA and its transcription factor FoxP3 mRNA expression in the sinonasal mucosa. IL-10 levels in the NLF were significantly increased in the CRS mouse model. These findings indicate that increased IL-10 in NLF may represent a compensatory immune homeostatic mechanism to counteract SEB and OVA-induced sinonasal mucosal inflammation. Moreover, intranasal administration of EI137 and EI341 appears to improve anti-inflammatory response through their immunomodulatory properties.

EI137 and EI341 effectively controlled SEB-induced sinonasal mucosal inflammation in the CRS mouse model, but several limitations were observed. First, CRS is a multifactorial disease with various causative factors, including viruses, fungi, and host immune status, whereas the SEB-induced CRS model is a well-established model for eosinophilic CRS. Therefore, the therapeutic efficacy of these lidocaine analogs in other CRS subtypes cannot be concluded. Second, intranasal instillation of lidocaine analogs demonstrated a systemic effect, as evidenced by the splenocyte study. However, there are several anatomical and immunological differences between mice and humans, and whether intranasal administration of these compounds would exert similar systemic effects in humans remains to be determined. Third, we assessed Th cytokines and their transcription factors to investigate the immunological effects of the lidocaine analogs; however, the detailed underlying mechanisms remain unclear and require further elucidation. Finally, the study was conducted with a relatively small experimental sample size. Further studies using a broader range of concentrations and various pathogenic stimuli are warranted to better characterize the immunological properties and therapeutic potential of EI137 and EI341.

## 4. Materials and Methods

### 4.1. Lidocaine-Derived Organic Compound Preparation

**Figure 6:**
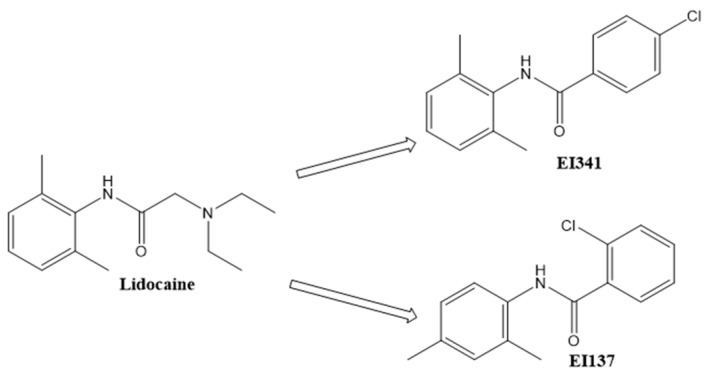
Structures of lidocaine-derived molecules.

EI137 and EI341 were prepared using established synthetic procedures at Assiut University (Assiut, Egypt) as part of a structure-based design focusing on lidocaine analogs (Figure 6) [19]. The design involved strategic lidocaine scaffold modifications. Design modifications targeted the methyl substitution pattern on the aromatic ring, altered the acyl group, and eliminated the tertiary amine related to sodium channel inhibition. Spectral and elemental analyses verified the structures and purity. EI137 and EI341 are positional isomers with identical molecular weights of 259.7 g/mol.

### 4.2. Development of SEB-Induced Eosinophilic CRS and the Experimental Protocol

Six-week-old female BALB/c mice (Hyosung Science Inc., Daegu, Republic of Korea), confirmed to be free of murine-specific pathogens, were used in this study. The animals were maintained under specific pathogen-free conditions in standard cages and were provided unrestricted access to food and water. All animal protocols were approved by the Institutional Review Board of Animal Experiments of Daegu Catholic University Medical Center (DCIAFCR-231205-30-YM; approved on 8 November 2024) and followed the guidelines of the National Institutes of Health.

On days 0, 7, 14, and 21, mice were sensitized by intraperitoneal injection of 75 μg of ovalbumin (OVA, Sigma-Aldrich, St. Louis, MO, USA) mixed with 2 mg of aluminum hydroxide. Subsequently, from days 22 to 28, they were challenged daily with intranasal administration of 3% OVA dissolved in 20 μL of phosphate-buffered saline (PBS). To maintain sinonasal inflammation, the mice were further challenged with OVA from week 5 to week 8. In addition to OVA, 10 ng/mL of SEB was administered intranasally once per week from 9 to 16 weeks. To assess the effects of lidocaine-derived organic compounds on CRS development, 0.5 or 5 μg/g of EI137 or EI341, respectively, was intranasally administered three times a week from week 9 to week 16. The mice were sacrificed on day 113 for further analysis (Figure S3). A total of 5 μg/g of lidocaine was utilized as a positive control, whereas PBS was applied as a negative control.

The experimental groups were designed as PBS instillation only (Group 1), OVA with 10 ng/mL of SEB instillation (Group 2), OVA with SEB treated with PBS (Group 3), OVA with SEB treated with 5 μg/g of lidocaine (Group 4), OVA with SEB treated with 0.5 μg/g of EI137 (Group 5), OVA with SEB treated with 5 μg/g of E137 (Group 6), OVA with SEB treated with 0.5 μg/g of EI341 (Group 7), and OVA with SEB treated with 5 μg/g of EI341 (Group 8). Each experimental group included a minimum of eight mice.

### 4.3. Evaluation of the Sinonasal Symptoms

The mice were allowed to acclimate in observation cages for about 10 min. After the final OVA exposure, nasal symptoms, including sneezing and nasal rubbing, were measured 5 min later over a 10 min interval. The outcomes were then compared among the experimental groups.

### 4.4. Assessment of Inflammatory Cells and Cytokines in NLF

NLF was obtained 24 h after the final administration of OVA. A 20-gauge catheter was gently inserted through a surgically exposed trachea into the nasopharyngeal space. The nasal cavity was washed with 1 mL of cold PBS, and the collected fluid was centrifuged at 2000 rpm for 7 min at 4 °C. The cell pellet was suspended in PBS, and a 10 μL aliquot was stained using May–Grunwald–Giemsa. Differential cell counts, including eosinophils, neutrophils, lymphocytes, and other leukocytes, were determined by averaging five high-power microscopic fields. The supernatant was stored at −70 °C for cytokine analysis (Interleukin (IL)-4, IL-10, interferon (IFN)-γ and tumor necrosis factor (TNF)-α) using commercially available enzyme-linked immunosorbent assay (ELISA) kits (R&D Systems, Inc., Minneapolis, MN, USA), with a detection limit of <2 pg/mL for each cytokine.

### 4.5. Measurement of Cytokine and Transcription Factor mRNA in the Sinonasal Mucosa

RNA was extracted from sinonasal tissues using TRIzol reagent (Invitrogen, Carlsbad, CA, USA) and its quality was assessed by spectrophotometry (FlUOstar Optima, BMG Labtech, Ortenberg, Germany). One microgram of RNA was used for cDNA synthesis using a reverse transcription thermal cycler (CFXOpus, Bio-Rad, Hercules, CA, USA). Quantitative PCR analysis was subsequently carried out using a SYBR Green-based PCR kit (PE Applied Biosystems, Foster City, CA, USA). The PCR primer sequence used in this study is listed in Table S1.

PCR cycling conditions were set as initial denaturation at 95 °C for 2 min, followed by 40 amplification cycles consisting of denaturation at 94 °C for 10 s, annealing at 60 °C for 10 s, and elongation at 72 °C for 45 s. Each reaction was run in two technical replications with three to four biological replicates. The 2^−ΔΔCT^ method was used to determine relative gene expression with β-actin serving as the reference gene for normalization.

### 4.6. Measurement of Cytokines from SEB-Stimulated Splenocytes

From each experimental group, splenocytes were obtained through mechanical dissociation of spleens and filtration using a 70 μm mesh. Red blood cells (RBCs) were eliminated by treatment with RBC lysis buffer. Cells were seeded in RPMI-1640 medium enriched with 10% fetal bovine serum, 100 U/mL penicillin, and 100 μg/mL streptomycin. A total of 5 × 10^6^ splenocytes were stimulated with 5 μg/mL SEB for 72 h. Cytokine concentrations (IL-4, IL-10, IFN-γ, and TNF-α) in the cultural supernatant were assessed using ELISA kits (R&D system).

### 4.7. Histological Evaluation of the Sinonasal Mucosa

Twenty-four hours after the final intranasal OVA challenge, mice were euthanized by intraperitoneal administration of pentobarbital sodium. Collected nasal tissues were decalcified in ethylenediaminetetraacetic acid and embedded in paraffin blocks. Serial coronal sections in 5 μm thickness were cut along the anteroposterior axis, and three anatomically matched sections were selected from each mouse as previously reported [20]. Hematoxylin-and-eosin staining was used to evaluate inflammatory cells infiltration and epithelial thickness, while periodic acid–Schiff (PAS) staining was used to quantify goblet cells. Inflammatory cell infiltration in submucosa was graded semi-quantitatively on a 0–30 scale (0: none; 10: mild; 20: moderate; 30: severe diffuse infiltration). Epithelial thickness was measured using a digital imaging system (Olympus Optical Co., Ltd., Tokyo, Japan), and analyzed using DP controller software (v2.2.1.227). Goblet cells were counted using an eyepiece reticle. All histological evaluations were conducted in a blinded manner, and average values were calculated from three mucosal regions per section across three sections per mouse.

### 4.8. Statistical Analysis

Data are expressed as the mean ± standard deviation, derived from eight independent replicates per experimental group. Student’s *t*-test was applied to compare two groups when data met the normality. In the case of multiple group comparisons with normally distributed variables, one-way analysis of variance (ANOVA) followed by Tukey’s multiple comparison test was used. For non-parametric data, the Mann–Whitney U test was employed for two-group comparisons, while the Kruskal–Wallis test followed by Bonferroni–Dunn post hoc correction was applied for comparison involving more than two groups. All statistical analysis was carried out using SPSS software (version 21.0; IBM Corp., Armonk, NY, USA). A *p*-value less than 0.05 was regarded as statistically significant.

## 5. Conclusions

The anti-inflammatory properties of the lidocaine-derived compounds EI137 and EI341 were investigated in a murine model of SEB-induced CRS. SEB stimulation led to elevated levels of IL-4, IL-10, and TNF-α in NLF, along with increased mRNA expression of these cytokines in the sinonasal mucosa. Intranasal administration of EI137 and EI341 markedly modulated cytokine production associated with Th2 and Treg responses, and alleviated SEB-induced sinonasal mucosal inflammation, characterized by immune cell infiltration and goblet cell hyperplasia. Notably, EI137 suppressed Th1 inflammation, whereas EI341 enhanced Th1 response in the sinonasal mucosa. These findings suggest that lidocaine analogs EI137 and EI341 may serve as promising topical therapeutic agents for Th2-dominant inflammatory diseases with distinct modulatory effects on Th1 immune response. However, for clinical application in humans, further studies are warranted to validate the efficacy of these compounds, including safety assessments using human cells and pharmacokinetic investigations.

## Data Availability

Data are contained within the article and Supplementary Materials; further inquiries can be directed to the corresponding author.

## Supplementary Materials

The following supporting information can be downloaded at https://www.mdpi.com/article/10.3390/ijms26178137/s1. Refs. [21,22,23] are cited in the Supplementary Materials.

## Abbreviations

The following abbreviations are used in this manuscript:

ANOVA	Analysis of variance
CRS	Chronic rhinosinusitis
ELISA	Enzyme-linked immunosorbent assay
IFN	Interferon
IL	Interleukin
NLF	Nasal lavage fluid
OVA	Ovalbumin
PAS	Periodic acid–Schiff
PBS	Phosphate buffered saline
RBC	Red blood cell
RT-PCR	Reverse-transcription polymerase chain reaction
SEB	Staphylococcal enterotoxin B
Th	T-helper
TNF	Tumor necrosis factor
