# Supplementary material for: Lidocaine-Based Derivatives for the Treatment of Staphylococcal Enterotoxin B-Induced Chronic Rhinosinusitis
Source: Int J Mol Sci. 2025 Aug 22;26(17):8137. doi: 10.3390/ijms26178137 (PMC12427834; doi:10.3390/ijms26178137)
Supplement: Supplementary file 1 [file ijms-26-08137-s001.zip › ijms-3718306-supplementary.pdf]

Method S1. Molecular docking study to determine interaction between lidocaine analogs and STAT-1.

The molecular docking studies were performed using MOE2020.09 software to investigate the binding interactions of EI137 and EI341 with STAT-1 (PDB: 1YVL). The STAT-1 protein structure was prepared by removing water molecules, adding hydrogen atoms, and optimizing protonation states at physiological pH (7.4). Both ligand structures (EI137 and EI341) were energy-minimized using the MMFF94x force field prior to docking.

The docking protocol employed the Triangular Matcher placement method followed by scoring with the London dG function to evaluate binding poses. To account for protein flexibility, we performed induced fit refinement of the top-scoring complexes. The docking grid was centered on STAT-1's phosphotyrosine binding pocket with a 20 Å cubic box dimension to ensure comprehensive sampling of potential binding sites.

For validation purposes, we redocked the native phosphopeptide ligand, achieving an acceptable RMSD of less than 2.0 Å compared to the crystallographic pose. The final complexes were analyzed based on binding energy scores and interaction patterns, with particular attention to key residues in the STAT-1 binding pocket. The top-ranked poses for each compound were selected for further analysis of their binding modes and potential biological implications.

References.

1. Trott, O.; Olson, A.J. AutoDock Vina: improving the speed and accuracy of docking with a new scoring function, efficient optimization, and multithreading. J Comput Chem 2010, 31, 455-461, doi:10.1002/jcc.21334.
2. Eberhardt, J.; Santos-Martins, D.; Tillack, A.F.; Forli, S. AutoDock Vina 1.2.0: New Docking Methods, Expanded Force Field, and Python Bindings. J Chem Inf Model 2021, 61, 3891-3898, doi:10.1021/acs.jcim.1c00203.
3. Laskowski, R.A.; Swindells, M.B. LigPlot+: multiple ligand-protein interaction diagrams for drug discovery. J Chem Inf Model 2011, 51, 2778-2786, doi:10.1021/ci200227u.

Result and Discussion S1. The interaction between lidocaine analogs and STAT-1.

Through molecular docking studies, we have elucidated the structural basis for the opposing biological activities of EI137 and EI341. The key findings reveal how these structurally similar compounds interact differently with STAT-1, leading to their distinct immunomodulatory effects (Figure S1 & S2).

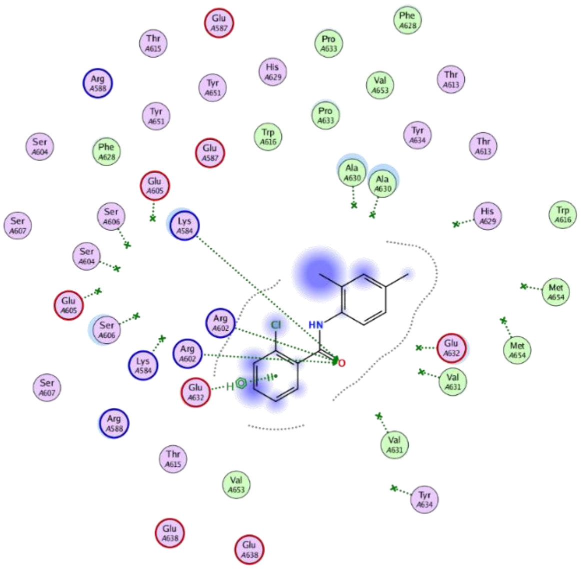

Figure S1. 2D interaction of EI137 with STAT-1

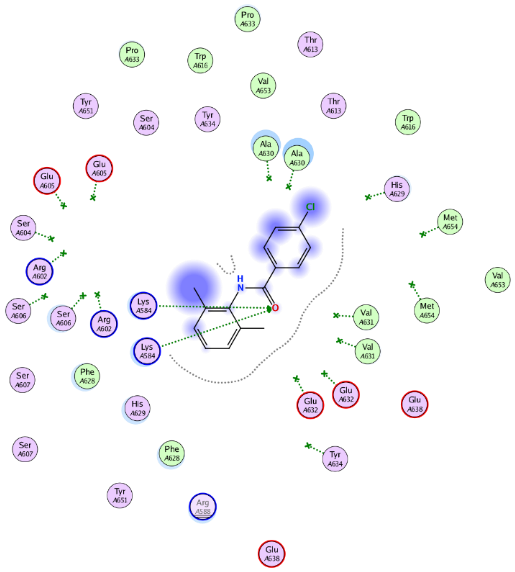

Figure S2. 2D interaction of EI341 with STAT-1

The docking results demonstrate that EI137 binds directly to the phosphotyrosine recognition site of STAT-1. The compound's amide carbonyl forms a critical hydrogen bond with Arg602, a residue essential for phosphotyrosine binding. This interaction is particularly significant as Arg602 plays a pivotal role in STAT-1 activation. By occupying this site, EI137 effectively blocks access to Tyr701, the phosphorylation site required for STAT-1 dimerization and subsequent activation. This binding mode provides a clear structural explanation for EI137's ability to suppress Th1 responses, as it directly interferes with the STAT-1 activation pathway.

In contrast, EI341 shows a markedly different interaction pattern. The compound positions itself adjacent to but not within the phosphotyrosine binding pocket. Notably, EI341's para-chloro orientation directs its amide group away from Arg602, preventing the formation of the key hydrogen bond observed with EI137. Instead, EI341 engages with nearby polar residues through weaker, non-competitive interactions. This alternative binding mode allows STAT-1 to maintain its activation capacity while potentially stabilizing certain conformational states that enhance Th1 responses.

The most striking observation is that despite their similar binding affinities (EI137: -5.16 kcal/mol; EI341: -5.07 kcal/mol), the two compounds produce opposite biological effects. This highlights that the specific nature of protein-ligand interactions, rather than binding affinity alone, determines functional outcomes. The ortho-chloro substitution in EI137 appears crucial for its inhibitory binding mode, while the para-chloro orientation in EI341 prevents this inhibitory interaction while still permitting STAT-1 engagement.

These computational findings correlate precisely with our experimental observations. The STAT-1-inhibitory binding of EI137 explains its suppression of IFN-γ production and Th1 responses, while EI341's non-competitive binding accounts for its Th1-enhancing activity. The results provide a clear example of how subtle structural modifications - particularly the position of chloro-substitution - can dramatically alter biological activity through specific changes in target interaction patterns.

From a therapeutic perspective, these insights demonstrate the potential for rational design of immunomodulators based on the lidocaine scaffold. The distinct binding modes of EI137 and EI341 suggest that this chemical framework can be modified to produce either Th1-suppressing or Th1-enhancing compounds, depending on the specific substitutions and their resulting interaction patterns with STAT-1.

While these docking studies provide valuable mechanistic insights, we acknowledge that further experimental validation would strengthen these conclusions. Future work could include biochemical assays to directly measure STAT-1 phosphorylation in the presence of these compounds, or structural studies to confirm the predicted binding modes. Nevertheless, the current findings offer a compelling structural explanation for the opposing biological effects of EI137 and EI341, addressing the fundamental question of how such similar compounds can produce distinct immunological outcomes.

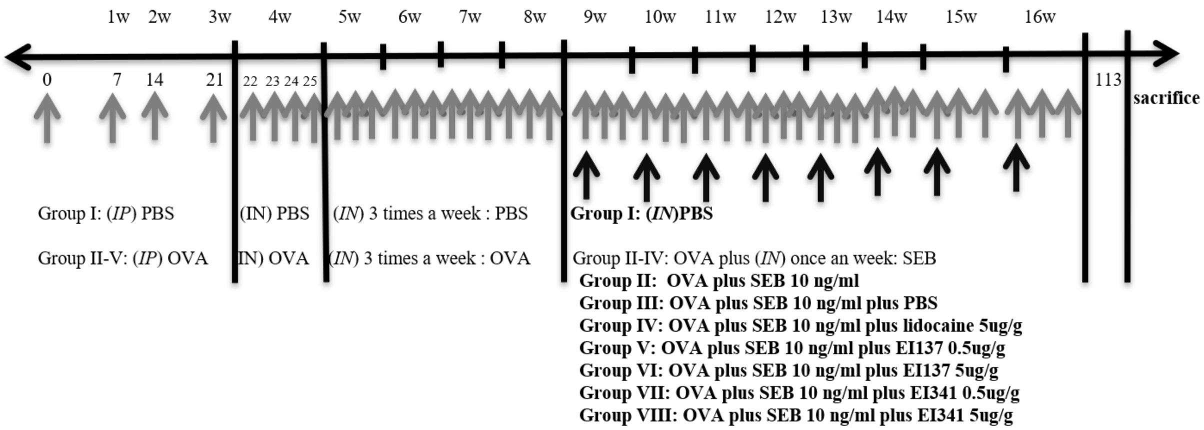

Figure S3. Schematic diagram of OVA-sensitized SEB induces an eosinophilic chronic rhinosinusitis mouse model. BV was applied 3 times a week from 9 weeks through 16 weeks. Gray arrows indicate lidocaine analogs administration, and black arrows indicate staphylococcal enterotoxin B administration. OVA: ovalbumin; SEB: Staphylococcal enterotoxin B; PBS: phospate-buffered saline; S, chronic rhinosinusitis; i.p., intraperitoneal; i.n., intranasally.

Table S1. Sequence of real-time polymerase chain reaction oligonucleotide primers.

Primer	Direction	Sequence	Size (bp)
IL-4	fowared	5′-CAATTGCAATGCCATCTACAGGAC-3′	104
	reverese	5′-TTTTGGTATCGGGGAGGCTG-3′	
IL-10	fowared	5′-GCCAGAGCCACATGCTCCTA-3′	145
	reverese	5′-GATAAGGCTTGGCAACCCAAGTAA-3′	
IFN-γ	fowared	5′-CGGCACAGTCATTGAAAGCCTA-3′	199
	reverese	5′-GTTGCTGATGGCCTGATTGTC-3′	
T-bet	fowared	5′-GCCAGGGAACCGCTTATA-3′	104
	reverese	5′-CCTTGTTGTTGGTGAGCTTTA-3′	
GATA-3	fowared	5′-TACCACCTATCCGCCCTATG-3′	101
	reverese	5′-GCCTCGACTTACATCCGAAC-3′	
Foxp3	fowared	5′-CACCTATGCCACCCTTATCCG-3′	91
	reverese	5′-CATGCGAGTAAACCAATGGTAGA-3′	
β-actin	fowared	5′-GCAGAAGGAGATTACTGCTCT-3′	136
	reverese	5′-GCTGATCCACATCTGCTGGAA-3′	
